# Early Electronic Screen Exposure and Autistic-Like Behaviors among Preschoolers: The Mediating Role of Caregiver-Child Interaction, Sleep Duration and Outdoor Activities

**DOI:** 10.3390/children7110200

**Published:** 2020-10-28

**Authors:** Jing-Yi Chen, Esben Strodl, Li-Hua Huang, Ying-Jie Chen, Gui-You Yang, Wei-Qing Chen

**Affiliations:** 1 Department of Epidemiology, School of Public Health, Sun Yat-sen University, Guangzhou 510080, China; chenjy246@mail2.sysu.edu.cn (J.-Y.C.); hlihua2@mail2.sysu.edu.cn (L.-H.H.); chenyj96@mail2.sysu.edu.cn (Y.-J.C.); yanggy7@mail2.sysu.edu.cn (G.-Y.Y.); 2 School of Psychology and Counselling, Queensland University of Technology, Brisbane 4059, Queensland, Australia; e.strodl@qut.edu.au; 3 Department of Information Management, Xinhua College of Sun Yat-sen University, Guangzhou 510080, China

**Keywords:** screen time, autistic-like behavior, caregiver-child interaction, sleep duration, outdoor activities, preschool children, mediation

## Abstract

Research into early screen exposure has raised growing concerns about its impact upon children’s neuropsychological well-being. However, possible pathways remain unclear. This study therefore aimed not only to evaluate the association between screen exposure during the ages of 0–3 years and preschoolers’ autistic-like behaviors, but also the mediating roles of the frequency of caregiver-child interaction, sleep duration and level of participation in outdoor activities. Based on the 2017 survey of the Longhua Child Cohort Study, data of 29,595 child-caregiver dyads were obtained via a caregiver-reported questionnaire, with the data from 29,461 dyads included in the data analysis. Multiple linear and logistic regression models were employed to estimate the associations between screen exposure, caregiver-child interaction, sleep duration, outdoor activities, and children’s autistic-like behaviors. The results indicated that screen exposure during 0–3 years of age was associated with the presence of autistic-like behaviors at preschool age, and the strength of the association was enhanced with the increase of average daily screen time (Odds Ratios (ORs) ranging from 1.358 to 4.026). The frequency of caregiver-child interaction and sleep duration mediated 5.32% and 1.19% of the variance of the association respectively, but outdoor activities did not mediate the association. Our findings indicate that preschoolers who are exposed to screens at aged 0–3 years might have an increased risk of autistic-like behaviors, and that, the frequency of caregiver-child interaction and sleep duration might function as potential mediators of this association.

## 1. Introduction

Currently, 1 in 160 children worldwide meet the criteria for autism spectrum disorder (ASD) [1], which is characterized by impairments in social interactions and communication, and restrictive and repetitive behavioral patterns [2]. Disabilities and comorbidities of ASD not only bring functional deficits to individuals, but also create a substantial burden for family and society due to the life-long demands of care and support [3,4]. Frequently, ASD goes undiagnosed until four years of age [5], while early emerging autistic-like behaviors such as eye fixations decline and avoidant responses to touch can be observed as early as 18–24 months [6,7,8]. A genetic overlap has been confirmed between individuals with ASD and with autistic-like behaviors [9], and delayed or atypical behaviors are associated with later ASD diagnosis [10,11]. As growing evidence supports that early behavioral intervention can improve core manifestations of ASD [12,13], the identification and the exploration of risk factors for the onset of autistic-like behaviors becomes increasingly consequential [14].

Although a variety of risk factors for ASD have been identified, none have been proven to be necessary or sufficient alone for ASD development [15,16]. With the rapid evolvement of screen-based technology, screen-based media has become a ubiquitous feature of early childhood. Younger ages of screen viewing, and early excessive screen exposure have become important environmental factors for children’s neuropsychological well-being [17,18]. For example, three reviews have respectively summarized the findings on screen-time-related adverse psychological outcomes among children and adolescents, such as depressive symptoms, attention problems, and poor cognitive development [18,19,20]. Regarding the adverse outcome of autistic-like behaviors, a cross-sectional survey among 8900 kindergarten children in Shanghai identified a positive relationship between screen time and the total score on the Clancy Autism Behavior Scale (CABS) [21]. A study by Must et al. showed that the average screen time on both weekdays and weekends among ASD children aged 3 to 11 years were approximately 1 h higher than children without any developmental disorders [22]. Another study by Chonchaiya et al. found that ASD children began to watch television around six months earlier than typically developing peers [23]. In addition, several case reports indicated that young autistic-like children with heavy screen use were re-assessed as non-autistic after a couple months of screen media isolation [24,25]. However, far little attention has been paid to the development of autistic-like behaviors due to early childhood screen exposure, especially at age 0–3 years, when brain plasticity and neurobehavioral development are at their peak [26,27,28].

Moreover, while the mechanisms explaining the aforementioned association remains unclear, several possible paths have been proposed [21]. Firstly, a study by Zhao et al. has indicated that caregiver-child interaction may play a mediating role in the association between screen time and prosocial behaviors [29]. This finding may be relevant to understanding a possible mechanism linking early screen exposure with the development of ASD, as low levels of prosocial behaviors are related to the core symptom of social skills impairment for children with ASD [30,31]. In addition, Wan et al. found that interactive caregiver–child communication was essential for shaping infant social competence because of the bi-directional influence of child and caregivers’ behavior upon each other [32]. Wan also demonstrated through a systematic review that the features of early caregiver-child interaction can be precursors to ASD [33]. This may be due to the frequency of parent-child verbal interaction as well as the quality of caregiver-child interaction being lowered as screen exposure increases [34,35]. Secondly, sleep duration has been found to mediate the association between screen time and psychological symptoms among early adolescents [36]. This mediating association could be due to the blue light emitted from electronic screens shortening children’s sleep duration by suppressing endogenous melatonin [37,38,39,40]. While insufficient sleep has been shown to predict later childhood adverse psychological outcomes such as attention deficit hyperactivity disorder, emotional problems and poor cognitive performance, the evidence for young children remains limited and so needs further investigation [41,42,43]. Thirdly, some researchers have found that longer screen time can lead to reduced outdoor activities [44]. Outdoor activities provide important opportunities to play and communicate with peers, which are in turn critical to the development of young children’s social skills [29,45]. It is therefore of interest to test whether the frequency of caregiver-child interaction, sleep duration and level of participation in outdoor activities may mediate the association between screen time and autistic-like behaviors, especially in a multiple mediator model [46]. Examining the possible mediating role of the above factors may contribute to the understanding of how screen exposure affects children’s neuropsychological development.

Given all of the above, the aim of our study was to explore the association between screen exposure at 0–3 years of age and the presence of preschoolers’ autistic-like behaviors, as well as to examine the extent that the frequency of caregiver-child interaction, sleep duration and the level of participation in outdoor activities mediate this association. These analyses provide further testing of the association between screen time and autistic like behaviors in early childhood, as well as providing preliminary exploration for potential mechanisms linking these variables.

## 2. Materials and Methods

### 2.1. Study Population

The participants were involved in the Longhua Child Cohort Study (LCCS) in 2017. This is an ongoing population-based child cohort study with once per year follow up since 2014. The study was designed to identify family and school risk and protective factors, during children’s early life, for subsequent psycho-behavioral development [47,48,49,50]. In 2017, a total of 29,595 caregivers of children aged 2 to 7 years completed the survey, and herein, 29,461 child-caregiver dyads were included for our analysis after excluding 134 participants with demographic information missing. Ethical approval was obtained from the Ethics Committee of the School of Public Health at Sun Yat-sen University (ethics clearance No.: 2015–016), and all caregiver participants gave fully informed written consent to participate in the study.

### 2.2. Data Collection

The data was collected from the children’s primary caregiver via a structured questionnaire. The questions asked about socio-demographic characteristics (including child’s age and gender, parents’ education level, marital status, age at child’s birth, and family monthly income) as well as autistic-like behaviors. The caregivers were also asked to recall the frequency and duration of children’s electronic screen exposure, caregiver-child interaction, outdoor activities, and children’s sleep duration during the period of 0–3 years retrospectively.

### 2.3. Measurement of Autistic-Like Behaviors

Autistic-like behaviors were assessed by the Autism Behavior Checklist (ABC) [51,52], a well-validated scale for ASD screening and diagnosis. The checklist was introduced in China by Yang et al. in 1993 [53], with an interrater reliability and test–retest reliability of 0.785 and 0.789, respectively, among Chinese children. The scale is comprised of five subscales (including sensory, relating, body and object use, language, and social and self-help skills) with a total of 57 items. Each item is weighted with score from 1 to 4, summing to obtain an overall score, with higher scores reflecting a greater probability of ASD. The recommended cut-offs for the Chinese version of the ABC were ≥31 points for screening and ≥62 points for diagnosis [53]. In order to increase sensitivity and avoid missing the borderline cases with autistic-like behaviors in mainstream kindergartens, a cut-off of ≥31 was selected in our study to distinguish children with and without autistic-like behaviors. We also repeated our analysis using cut-off of ≥62 to verify our assumption.

### 2.4. Measurement of Electronic Screen Exposure

Electronic screen exposure was measured according to a set of questions answered by the primary caregiver, who was asked to indicate how long on average his/her child spent viewing screens every day (including television, mobile phones, tablet, video games, etc.). These questions were repeated every year since child’s birth. Response options were categorized into 0 (none), 1 (<30 min/day), 2 (30–60 min/day), 3 (60–90 min/day), 4 (90–120 min/day), and 5 (>120 min/day). The specific year when a child started to have electronic screen assessment was defined as the initial age of screen exposure, and the total years with screen exposure was counted to yield the cumulative years of exposure. Screen time for each year was summed and divided by cumulative years to obtain the average screen time, which was then converted into 4 levels (none, <60 min/day, 60–120 min/day and >120 min/day) [47,54].

### 2.5. Measurement of Caregiver-Child Interaction

The frequency of caregiver-child interaction was determined by a series of questions: (1) How often did the caregiver perform the following specific activities with his/her child at aged 0 to 1 years: singing, chatting, playing games, body touch and outdoor activities, respectively; (2) How often did the caregiver perform the following specific activities with his/her child at aged 1 to 3 years: reading, singing, chatting, playing games, body touch, going to parties, outdoor activities and travelling, respectively. The assessment of frequency was operationalized into 5 rated categories: 0 (never), 1 (<1 time per week), 2 (1–2 times per week), 3 (3–6 times per week), 4 (every day). Scores for each item were added to obtain a total score, which was then divided by the number of items to yield the average frequency of caregiver-child interaction at aged 0–3 years [49,50].

### 2.6. Measurement of Sleep Duration

Children’s typical duration, in hours per night, at aged 0–3, 4–6, 7–12 months, and 1–3 years was reported by their primary caregivers. Sleep duration of the above periods was added up and the mean value calculated to estimate children’s average sleep duration at aged 0–3 years old.

### 2.7. Measurement of Outdoor Activities

The frequency and duration of children’s outdoor activities at aged 0–1, 1–2 and 2–3 years were reported by children’s primary caregivers separately. Options for frequency included 0 (never), 1 (1–2 times/week), 2 (3–4 times/week), 3 (5–6 times/week), 4 (7–8 times/week), 5 (9–10 times/week), 6 (11–12 times/week), 7 (>13 times/week), and options for duration included 0 (none), 1 (<30 min/day), 2 (30–60 min/day), 3 (60–90 min/day), 4 (90–120 min/day), and 5 (>120 min/day). The product of frequency and duration of each year was computed and summed to obtain an index for assessing the children’s level of participation in outdoor activities at aged 0–3 years.

### 2.8. Covariates

Based on previous studies [29,49,50], the following variables were included as covariates in the regression models: child’s age and gender, parents’ education level, marital status, age at child’s birth, and family monthly income.

### 2.9. Statistical Analysis

**Figure 1:**
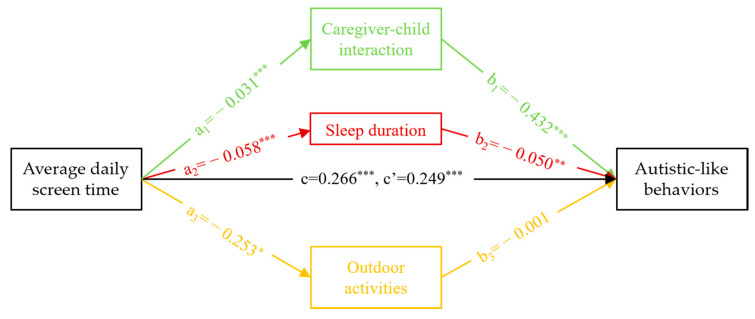
Multiple mediation model between average daily screen time on autistic-like behaviors through caregiver-child interaction, sleep duration and outdoor activities. Coefficients are shown after adjusting for child’s age, child’s gender, maternal and paternal age at child’s birth, maternal and paternal education level, monthly household income, parental marital status. The letter a_1_, a_2_ and a_3_ represented the coefficient of average daily screen time in the linear regression model to caregiver-child interaction, sleep duration and outdoor activities respectively; The letter b_1_, b_2_ and b_3_ represented the coefficient of caregiver-child interaction, sleep duration and outdoor activities in the logistic model to Autistic-like behaviors respectively; The letter c represented the coefficient of average daily screen time in the logistic model to Autistic-like behaviors without the effect of caregiver-child interaction, sleep duration and outdoor activities; The letter c’ represented the coefficient of average daily screen time in the logistic model to Autistic-like behaviors introducing the effect of caregiver-child interaction, sleep duration and outdoor activities. * *p* < 0.05; ** *p* < 0.01; *** *p* < 0.001.

Means (with standard deviation) for continuous variables, and frequencies (with proportions) for categorical variables were presented to describe the socio-demographic characteristics of the participants. Difference in these variables between children with and without autistic-like behaviors were compared using Chi-square tests and Student’s *t*-tests. We conducted a series of linear regression models and logistic regression models to examine the associations among screen exposure at age 0 to 3 years, the frequency of caregiver-child interaction, sleep duration, the level of participation in outdoor activities, and autistic-like behaviors. Furthermore, to verify whether, and the extent that, the frequency of caregiver-child interaction, sleep duration and level of outdoor activities mediated the association between average daily screen time and autistic-like behaviors, a multiple mediation model (Figure 1) was applied using a 5000 resampling bootstrapping procedure employing PROCESS macro for Statistical Package for Social Sciences (SPSS) [55,56]. Effects were estimated with bias corrected bootstrap 95% confidence intervals (CI), which were considered significant if the upper and lower bound of the 95% CI did not straddle zero. All of the regression analyses were adjusted for the aforementioned covariates. Data management and statistical analysis were carried out with SPSS (version 25.0; SPSS Inc., Chicago, IL, USA), and statistical significance was accepted as a two-tailed test with *p* < 0.05.

## 3. Results

### 3.1. Social-Demographic Characteristics of Participants by Status of Autistic-Like Behaviors

Of the 29,461 children investigated, 875 (2.97%) were categorized with autistic-like behaviors. The mean age of children with and without autistic-like behaviors were 4.33 ± 0.89 and 4.60 ± 0.88 years, respectively. Significant differences were found in child’ gender, parents’ age at birth, parents’ education level, marital status, and monthly household income between children with and without autistic-like behaviors. See more details in Table 1. Similar results were found when using 62 as the cut-off, and these results are displayed in Table S1 of the Supplementary Materials.

### 3.2. Associations between Electronic Screen Exposure at Age 0 to 3 Years and Autistic-Like Behaviors in Preschoolers

Table 2 presents the associations between electronic screen exposure and autistic-like behaviors as assessed using logistic regression models adjusted by the aforementioned covariates. Compared with children without screen exposure at aged 0–3 years, those with past exposure to electronic screens had a higher risk of autistic-like behaviors (Adjusted Odds Ratio (AOR) = 1.900, 95% CI = 1.551~2.327). The AOR of exhibiting autistic-like behaviors was 1.358 (95% CI = 1.071~1.722) for <30 min/day, 1.802 (95% CI = 1.429~2.273) for 30–60 min/day, 1.967 (95% CI = 1.521~2.543) for 60–90 min/day, 2.982 (95% CI = 2.291~3.882) for 90–120 min/day, and 4.026 (95% CI = 3.009~5.387) for >120 min/day of children’s average screen time compared with those who had never been exposed to an electronic screen. Similar impacts were suggested when using 62 as the cut-off, and the results can be found in Table S2 of the Supplementary Materials.

### 3.3. Associations between Average Daily Screen Time, Caregiver-Child Interaction, Sleep Duration, Outdoor Activities and Autistic-Like Behaviors

After controlling for the aforementioned covariates, linear regression analysis indicated that average daily screen time was negatively associated with the frequency of caregiver-child interactions (β = −0.031, 95% CI = −0.037~−0.026), sleep duration (β = −0.058, 95% CI = −0.075~−0.040), and the level of participation in outdoor activities (β = −0.253, 95% CI = −0.469~−0.037), respectively; while the logistic regression analysis showed that autistic-like behaviors were negatively related to caregiver-child interaction (AOR = 0.621, 95% CI = 0.567~0.679), sleep duration (AOR = 0.928, 95% CI = 0.899~0.958), and outdoor activities (AOR = 0.995, 95% CI = 0.992~0.998) respectively. See more details in Table 3. A similar trend was observed when using 62 as the cut-off, and the results are shown in Table S3 of the Supplementary Materials.

### 3.4. Mediation Effect of Average Daily Screen Time on Autistic-Like Behaviors through Caregiver-Child Interaction, Sleep Duration and Outdoor Activities

As illustrated in Table 4 and Figure 1, the direct and total indirect effect of average daily screen time on autistic-like behaviors were respectively 0.249 (95% CI = 0.203~0.295) and 0.017 (95% CI = 0.013~0.021). The bias-corrected bootstrap 95% CI indicated that the indirect effect of the frequency of caregiver-child interaction (effect size = 0.014, 95% CI = 0.010~0.018) and sleep duration (effect size = 0.003, 95% CI = 0.001~0.005) were significant, which mediated 5.32% and 1.19% of the association respectively. No significant mediating effect for the level of participation in outdoor activities was found. Moreover, contrasting the effect size among the mediators, the frequency of caregiver-child interaction had the greatest indirect effect, following by sleep duration, and then the level of participation in outdoor activities. Similar results when using the cut-off of 62 are shown in Table S4 of the Supplementary Materials.

## 4. Discussion

Based on the cross-sectional survey data from the LCCS in 2017, we explored the associations between screen exposure, the frequency of caregiver-child interaction, sleep duration, level of participation in outdoor activities in early life (aged 0–3 years) and autistic-like behaviors at preschool age. The results indicated that early screen exposure at aged 0–3 years was associated with the presence of autistic-like behaviors at preschool age with the strength of the association being enhanced with the increase of average daily screen time. In addition, the frequency of caregiver-child interactions and sleep duration during the first three years of life mediated 5.32% and 1.19% of the variance of the association, respectively.

### 4.1. Association between Early Electronic Screen Exposure and Autistic-Like Behaviors in Preschoolers

It has been well documented that excessive screen time can cause adverse psychological effects including depressive symptoms, attention problems, and poor cognitive development, in adolescents and adults [18,19,20]. Regarding the association between electronic screen exposure and ASD, two case-control studies respectively found that ASD children had longer average screen time [22] and started the screen viewing at a younger age than typically developing peers [23]. Recently, a report showed that symptoms in young ASD patients exposed to screen media heavily (>4 h/day) from a very young age in several countries (e.g., France, Japan, USA, Thailand, etc.) significantly declined after screen removal intervention for a few months [24]. Moreover, a cross-sectional study among 8900 preschoolers aged 3–6 in China indicated that children exposed to screens ≥2 h/day significantly increased the risk of ASD behavioral symptoms [21]. In line with the above findings, the present study also observed that early electronic screen exposure at aged 0–3 years was associated with the presence of autistic-like behaviors at preschool age, and the risk was significantly enhanced with the increase of average daily screen time. All these findings support the American Academy of Pediatrics (AAP) [57], the World Health Organization (WHO) [58] and the Canadian Paediatric Society [59] recommendations that children prior to two years of age should be limited in any form of screen exposure.

### 4.2. Association between Early Electronic Screen Exposure and Caregiver-Child Interaction, Sleep Duration, and Outdoor Activities

A negative association was observed between average screen time and the frequency of caregiver-child interaction, sleep duration, and level of participation in outdoor activities in our study. This negative association might be explained by the theory of time displacement, in that screen time could replace the time which was supposed to be spent on other activities [60,61]. Our findings are consistent with previous research. Firstly, a study by Krupa et al. found an overall negative association between family screen time and the duration of caregiver-child reciprocal interaction [62]. Similarly, a study by Mendelsohn et al. showed that verbal interactions between mothers and children were limited during screen exposure, with only 42.8% mothers talking to their children while co-viewing educational programs and 21.3% during non-educational programs [63]. Moreover, a prospective study showed that increasing mother-child interaction at 18 months could reduce children’s screen time at subsequent two and three years of age [64]. These findings are similar to our results, showing a negative association between screen time and the frequency of caregiver-child interaction. Secondly, longer average screen time was shown to be associated with the reduction of sleep duration among preschoolers in our study. Consistent with this, Twenge et al. found that using portable and nonportable electronic devices over 2 h per day was significantly related to sleep insufficiency among children under five years old in the USA [65]. In addition, Hu et al. reported that with each additional hour of screen time, 3 min of shorter sleep, 1.6 min of longer sleep latency and 4 min of later bedtime were found among children two–five years old in Sydney [66]. Thirdly, it has been well proved that higher screen time is related to children’s lower levels of physical activity [44,67,68] and that early exposure to screen devices is negatively associated with children’s later outdoor playtime at preschool age [69]. These findings concur with our findings of a negative association between screen time and the frequency and duration of outdoor activities.

### 4.3. Associations of Caregiver-Child Interaction, Sleep Duration, and Outdoor Activities with Autistic-Like Behaviors in Preschoolers

The frequency of caregiver-child interaction was inversely associated with autistic-like behaviors in our study. This is consistent with previous studies’ showing that caregiver-child interaction is critical for children’s neurobehavioral development, especially for language and executive function development [35,70,71,72,73]. Recently, Parent-Child Interaction Therapy (PCIT) has shown to significantly improve ASD children’s disruptive behavior, externalizing behavior and executive function, which provided clues for the positive effect of caregiver-interaction on autistic symptoms [74,75]. In addition, we found that sleep duration was negatively related to autistic-like behaviors, being in line with prior investigations reporting that children with shorter sleep duration had higher scores on the Clancy Autism Behavior Scale (CABS), indicating an increased risk of developing ASD related behavioral problems [21]. Furthermore, it has been found that sleep duration shorter than 10 h per day at 18 months predict the subsequent emotional and behavioral problems including anxious/depressed states, attention problems, aggressive behaviors [76]. Although few previous investigations have discussed the association between outdoor activities and children’s autistic-like behaviors, we observed a weak negative association between these variables. However, Hinkley et al. found that outdoor play was favorably associated with good social skills among preschoolers [45], and an outdoor intervention implemented among ASD children resulted in improvement in their autistic mannerisms [77].

### 4.4. Potential Pathways for Early Electronic Screen Exposure Causing Autistic-Like Behaviors in Preschoolers

Unfortunately to-date few researchers have probed into the underlying mechanisms linking screen exposure with children’s neuropsychological disorders, and so the evidence still remains limited. For example, Zhao et al. demonstrated that parent-child interaction mediated the effect of excessive screen time on children’s psychosocial well-being, explaining 28.1% of the effect on total difficulties and 58.6% on prosocial behavior among children aged 3–4 years [29]. Guerrero et al. found that sleep duration mediated 0.2%–1.0% of the relationship between screen time and problem behaviors in children aged 9 to 10 years [61]. Li et al. observed that sleep accounted for 38.5% of the association between adolescent screen time and depressive symptoms [78]. Analogously, we observed that the frequency of caregiver-child interaction and sleep duration acted as significant mediators of the impact of early screen time on children’s autistic-like behaviors explaining 5.32% and 1.19% of the total effect, respectively. Our results showed that screen time affected the caregiver-child interaction most strongly among these three mediating factors. A possible reason for the weak mediating effect of sleep in our current and two previous studies [29,61], might be that very young children are usually exposed to screens during day time when they are awake rather than prior to their bedtime at night [29,79]. That is, perhaps the duration between screen exposure and sleep mitigated the mediating effect of sleep between young children’s screen exposure and their neuropsychological disorders (in this case autistic-like behaviors). The mediating effect of the level of participation in outdoor activities was insignificant in our study. We suspect that children under three years old are still too young to engage in significant levels of peer communication and teamwork learning associated with many childhood outdoor activities [80], but further investigation is needed. Furthermore, given the total effect size of the mediation model was modest, accounting for a mere 6.39% of the association, other potential mechanisms are called for investigation in future research. Possible mediators to explore in future research include sleep-related mediating variables such as sleep quality, sleep disturbance and sleep variability [61,81], the specific neurotransmitter deficiency due to excessive screen use [82], lower integrity of the brain white matter tracts (supporting language and literacy skills) linked to increased screen time [83], and competence in social and non-social sensory processing caused by screen-based audiovisual input in infancy [84].

### 4.5. Strength and Limitation

To the best of our knowledge, this was the first study that investigated the association between early childhood electronic screen exposure and the presence of autistic-like behaviors. It is also the first study to explore the mediating roles of frequency of caregiver-child interaction, sleep duration, and the level of participation in outdoor activities using a multiple mediator model. A major strength of this study was the analysis of a large sample of Chinese preschoolers. However, several limitations deserve consideration when interpreting the results of our study. First, the cross-sectional design limited the ability to make any causal inferences on the direction of the relationships. Results from the present study should therefore be interpreted cautiously, and longitudinal research is warranted to further verify the causality and mediation effect. Second, all participants in our study were recruited in Longhua District in Shenzhen. The prevalence of ASD in Longhua District, Shenzhen was 2.6% according to a mainstream kindergarten-based investigation of suspected autism in 2014 [85], while the pooled prevalence of ASD among China was 0.39% [86]. As such, there is the possibility of selection bias that lowers the generalizability of the findings. Third, data about screen use, caregiver-child interaction, sleep, and outdoor activities at the period of 0–3 years were asked of parents when their child was between 4–7 years. As such there is the possibility of recall bias on account of the retrospectively reported measurements. The interpretation of the results therefore requires caution and caveats. The findings from this study need to be replicated using more valid and reliable measures of screen use, caregiver-child interaction, sleep, and outdoor activities using, for example, daily records monitored by electronic devices. Fourth, family history of mental health problems was unavailable in our dataset and so was not controlled for in the analyses. It is possible that this may act as a potential confounder of the above findings. Fifth, we excluded 134 participants with incomplete information, whose demographic characteristic on child’s age, paternal age at child’s birth, monthly household income and parental marital status were found different to those we enrolled (Table S5 in the Supplementary Materials compares the social-demographic characteristics and study variables among population enrolled and excluded). Although this was a small proportion of our study population and we have adjusted for the above variables in our regression model, selection bias might still have existed. Sixth, the details of screen exposure and caregiver-child interaction were not comprehensive. For example, only the frequency and duration of screen exposure were assessed without considering the content of screen exposure and parents’ co-viewing [29,87]. Similarly, only the frequency of caregiver-child interaction was measured without collecting the details of duration and quality. Therefore, further investigations with more comprehensive evaluation of the variables are necessary to provide a more precise and sophisticated examination of the identified association.

## 5. Conclusions

Our study showed that early screen exposure at aged 0–3 years was associated with the increased occurrence of autistic-like behaviors at preschool age. In addition, the frequency of caregiver-child interaction and sleep duration during early childhood were found to be mediators of this association. Such findings extend the evidence for the adverse effect of early childhood screen exposure on young children’s neuropsychological development and offer a preliminary explanation for potential mechanisms. However, limited by the cross-sectional design and the retrospective reports, longitudinal studies are required to verify the causal link between early childhood screen exposure and the later development of autistic-like behaviors. Moreover, other underlying mechanisms need to be investigated to provide a more comprehensive explanation for why and how the effects occur. This line of research is important in guiding tailored recommendations on healthier screen use for young children.

## Figures and Tables

**Table 1:** Social-demographic characteristics of the participants by status of autistic-like behaviors.

Characteristics	Total (*N* = 29,461)	Autistic-Like Behaviors		χ^2^/t	*p*-Value
		No (*N* = 28,586)	Yes (*N* = 875)		
Child’s age [mean ± SD (years)]	29,461	4.60 ± 0.88	4.33 ± 0.89	8.98	<0.001
Maternal age at child’s birth [mean ± SD (years)]	29,461	27.17 ± 4.22	26.01 ± 4.52	7.46	<0.001
Paternal age at child’s birth [mean ± SD (years)]	29,461	29.73 ± 4.81	28.80 ± 5.07	5.36	<0.001
Child’s gender [n (%)]				22.46	<0.001
Male	16,000	15,456 (54.1)	544 (62.2)		
Female	13,461	13,130 (45.9)	331 (37.8)		
Maternal education level [n (%)]				95.78	<0.001
Junior high school or lower	7367	7041 (24.6)	326 (37.3)		
High school	8604	8333 (29.2)	271 (31.0)		
College	7236	7073 (24.7)	163 (18.6)		
Undergraduate or above	6254	6139 (21.5)	115 (13.1)		
Paternal education level [n (%)]				114.61	<0.001
Junior high school or lower	6076	5787 (20.2)	289 (33.0)		
High school	7954	7689 (26.9)	265 (30.3)		
College	6777	6624 (23.2)	153 (17.5)		
Undergraduate or above	8654	8486 (29.7)	168 (19.2)		
Monthly household income [n (%)]				91.15	<0.001
≤¥5000 Yuan	4341	4150 (14.5)	191 (21.8)		
¥5000–10,000 Yuan	7779	7487 (26.2)	292 (33.4)		
¥10,001–15,000 Yuan	5645	5479 (19.2)	166 (19.0)		
¥15,001–20,000 Yuan	4083	4006 (14.0)	77 (8.8)		
>¥20,000 Yuan	7613	7476 (26.1)	149 (17.0)		
Parental marital status [n (%)]				16.67	<0.001
Married	28,417	27,595 (96.5)	822 (93.9)		
Unmarried/Divorced/ Widowed/Remarried	1044	991 (3.5)	53 (6.1)		

χ^2^: Value for Chi-square test. t: Value for Student’s t-test. SD: Standard Deviation. n (%): Number (proportion).

**Table 2:** Associations between electronic screen exposure at age 0 to 3 years and autistic-like behaviors in preschoolers.

Screen Exposure at Age 0 to 3 Years	Autistic-Like Behaviors (*N* = 29,461)		
	Number of Children	Cases (*N*%)	AOR (95% CI)
Exposure to electronic screens			
No	7097	112 (1.6)	Ref
Yes	22,364	763 (3.4)	1.900 (1.551, 2.327) ***
Average daily screen time (minutes)			
Never	7097	112 (1.6)	Ref
<30	8271	190 (2.3)	1.358 (1.071, 1.722) *
30–60	6857	223 (3.3)	1.802 (1.429, 2.273) ***
60–90	3723	137 (3.7)	1.967 (1.521, 2.543) ***
90–120	2303	125 (5.4)	2.982 (2.291, 3.882) ***
>120	1210	88 (7.3)	4.026 (3.009, 5.387) ***

Adjusted for child’s age, child’s gender, maternal and paternal age at child’s birth, maternal and paternal education level, monthly household income, parental marital status. AOR: Adjusted odds ratio. CI: Confidence intervals. Ref: Reference. * *p* < 0.05; *** *p* <0.001.

**Table 3:** Associations between average daily screen time, caregiver-child interaction, sleep duration, outdoor activities, and autistic-like behaviors.

	Average Daily Screen Time at Age 0 to 3 Years (Minutes)	Autistic-Like Behaviors
	β, 95%CI	AOR, 95%CI
Caregiver-child interaction	−0.031 (−0.037, −0.026) ***	0.621 (0.567, 0.679) ***
Sleep duration	−0.058 (−0.075, −0.040) ***	0.928 (0.899, 0.958) ***
Outdoor activities	−0.253 (−0.469, −0.037) *	0.995 (0.992, 0.998) ***

Adjusted for child’s age, child’s gender, maternal and paternal age at child’s birth, maternal and paternal education level, monthly household income, parental marital status. β: The coefficient of linear regression models. CI: Confidence intervals. AOR: Adjusted odds ratio. * *p* < 0.05; *** *p* < 0.001.

**Table 4:** Mediation effect of average daily screen time on autistic-like behaviors through caregiver-child interaction, sleep duration and outdoor activities.

	Effect Size (SE)	Bootstrapping BC 95% CI		Proportion of Indirect Effect
		Lower	Upper	
	Direct effects			
	0.249 (0.023)	0.203	0.295	
	Indirect effects			
Caregiver-child interaction	0.014 (0.002)	0.010	0.018	5.32%
Sleep duration	0.003 (0.001)	0.001	0.005	1.19%
Outdoor activities	0.000 (0.000)	0.000	0.002	NS
Total	0.017 (0.002)	0.013	0.021	6.39%
	Contrasts			
Caregiver-child interaction vs. sleep duration	0.011 (0.002)	0.006	0.015	
Caregiver-child interaction vs. outdoor activities	0.013 (0.002)	0.009	0.018	
Sleep duration vs. outdoor activities	0.003 (0.001)	0.001	0.005	

A bootstrapping procedure with 5000 resamples was implemented by the PROCESS macro for SPSS. Indirect effects were estimated with bias corrected confidence intervals (BC 95% CI), which were considered significant if the upper and lower bound of the 95% CI did not straddle zero. NS: Not significant. Adjusted for child’s age, child’s gender, maternal and paternal age at child’s birth, maternal and paternal education level, monthly household income, parental marital status.

## Supplementary Materials

The following are available online at https://www.mdpi.com/2227-9067/7/11/200/s1, Table S1: Social-demographic characteristics of the participants by status of Autistic-like behaviors (62 as the cut-off), Table S2: Associations between electronic screen exposure at age 0 to 3 years and autistic-like behaviors in preschoolers (62 as the cut-off), Table S3: Associations among average daily screen time, caregiver-child interaction, sleep duration, outdoor activities and autistic-like behaviors (62 as the cut-off), Table S4: Mediation effect of average daily screen time on autistic-like behaviors through caregiver-child interaction, sleep duration and outdoor activities (62 as the cut-off), Table S5: Comparison of social-demographic characteristics and study variables among population enrolled and excluded.

## References

[1] Elsabbagh M., Divan G., Koh Y.J., Kim Y.S., Kauchali S., Marcin C., Montiel-Nava C., Patel V., Paula C.S., Wang C. (2012). Global prevalence of autism and other pervasive developmental disorders. Autism Res..

[2] American Psychiatric Association (2013). Diagnostic and Statistical Manual of Mental Disorders.

[3] Lord C., Elsabbagh M., Baird G., Veenstra-Vanderweele J. (2018). Autism spectrum disorder. Lancet.

[4] Buescher A.V., Cidav Z., Knapp M., Mandell D.S. (2014). Costs of autism spectrum disorders in the United Kingdom and the United States. JAMA Pediatr..

[5] Zwaigenbaum L., Penner M. (2018). Autism spectrum disorder: Advances in diagnosis and evaluation. BMJ.

[6] Yoon J.M., Vouloumanos A. (2014). When and how does autism begin?. Trends Cogn. Sci..

[7] Mammen M.A., Moore G.A., Scaramella L.V., Reiss D., Ganiban J.M., Shaw D.S., Leve L.D., Neiderhiser J.M. (2015). Infant Avoidance during a Tactile Task Predicts Autism Spectrum Behaviors in Toddlerhood. Infant Ment Health J.

[8] Elsabbagh M. (2020). Linking risk factors and outcomes in autism spectrum disorder: Is there evidence for resilience?. BMJ.

[9] Bralten J., van Hulzen K.J., Martens M.B., Galesloot T.E., Arias Vasquez A., Kiemeney L.A., Buitelaar J.K., Muntjewerff J.W., Franke B., Poelmans G. (2018). Autism spectrum disorders and autistic traits share genetics and biology. Mol. Psychiatry.

[10] Karp E.A., Ibanez L.V., Warren Z., Stone W.L. (2017). Brief Report: What Drives Parental Concerns About Their 18-Month-Olds at Familial Risk for Autism Spectrum Disorder?. J. Autism Dev. Disord..

[11] Pijl M.K.J., Bussu G., Charman T., Johnson M.H., Jones E.J.H., Pasco G., Oosterling I.J., Rommelse N.N.J., Buitelaar J.K., Team B. (2019). Temperament as an Early Risk Marker for Autism Spectrum Disorders? A Longitudinal Study of High-Risk and Low-Risk Infants. J. Autism Dev. Disord..

[12] Reichow B., Hume K., Barton E.E., Boyd B.A. (2018). Early intensive behavioral intervention (EIBI) for young children with autism spectrum disorders (ASD). Cochrane Database Syst. Rev..

[13] Dawson G., Rogers S., Munson J., Smith M., Winter J., Greenson J., Donaldson A., Varley J. (2010). Randomized, controlled trial of an intervention for toddlers with autism: The Early Start Denver Model. Pediatrics.

[14] Sacrey L.R., Zwaigenbaum L., Bryson S., Brian J., Smith I.M., Roberts W., Szatmari P., Vaillancourt T., Roncadin C., Garon N. (2018). Parent and clinician agreement regarding early behavioral signs in 12- and 18-month-old infants at-risk of autism spectrum disorder. Autism Res..

[15] Lai M.-C., Lombardo M.V., Baron-Cohen S. (2014). Autism. Lancet.

[16] Bolte S., Girdler S., Marschik P.B. (2019). The contribution of environmental exposure to the etiology of autism spectrum disorder. Cell. Mol. Life Sci..

[17] Przybylski A.K., Weinstein N. (2019). Digital Screen Time Limits and Young Children’s Psychological Well-Being: Evidence From a Population-Based Study. Child Dev..

[18] Lissak G. (2018). Adverse physiological and psychological effects of screen time on children and adolescents: Literature review and case study. Environ. Res..

[19] Stiglic N., Viner R.M. (2019). Effects of screentime on the health and well-being of children and adolescents: A systematic review of reviews. BMJ Open.

[20] Carson V., Kuzik N., Hunter S., Wiebe S.A., Spence J.C., Friedman A., Tremblay M.S., Slater L.G., Hinkley T. (2015). Systematic review of sedentary behavior and cognitive development in early childhood. Prev. Med..

[21] Wu X., Tao S., Rutayisire E., Chen Y., Huang K., Tao F. (2017). The relationship between screen time, nighttime sleep duration, and behavioural problems in preschool children in China. Eur. Child Adolesc. Psychiatry.

[22] Must A., Phillips S.M., Curtin C., Anderson S.E., Maslin M., Lividini K., Bandini L.G. (2014). Comparison of sedentary behaviors between children with autism spectrum disorders and typically developing children. Autism.

[23] Chonchaiya W., Nuntnarumit P., Pruksananonda C. (2011). Comparison of television viewing between children with autism spectrum disorder and controls. Acta Paediatr..

[24] Harle B. (2019). Intensive early screen exposure as a causal factor for symptoms of autistic spectrum disorder: The case for <<Virtual autism>>. Trends Neurosci. Educ..

[25] Numata-Uematsu Y., Yokoyama H., Sato H., Endo W., Uematsu M., Nara C., Kure S. (2018). Attachment Disorder and Early Media Exposure: Neurobehavioral symptoms mimicking autism spectrum disorder. J. Med. Investig..

[26] Lane R., Radesky J. (2019). Digital Media and Autism Spectrum Disorders: Review of Evidence, Theoretical Concerns, and Opportunities for Intervention. J. Dev. Behav. Pediatr..

[27] Wen X., Zhang H., Li G., Liu M., Yin W., Lin W., Zhang J., Shen D. (2019). First-year development of modules and hubs in infant brain functional networks. Neuroimage.

[28] Jones R.A., Hinkley T., Okely A.D., Salmon J. (2013). Tracking physical activity and sedentary behavior in childhood: A systematic review. Am. J. Prev. Med..

[29] Zhao J., Zhang Y., Jiang F., Ip P., Ho F.K.W., Zhang Y., Huang H. (2018). Excessive Screen Time and Psychosocial Well-Being: The Mediating Role of Body Mass Index, Sleep Duration, and Parent-Child Interaction. J. Pediatr..

[30] Van Hoorn J., Van Dijk E., Crone E., Stockmann L., Rieffe C. (2017). Peers Influence Prosocial Behavior in Adolescent Males with Autism Spectrum Disorders. J. Autism Dev. Disord..

[31] Teng B., Nonneman R., Agster K., Nikolova V., Davis T., Riddick N., Baker L., Pedersen C., Jarstfer M., Moy S. (2013). Prosocial effects of oxytocin in two mouse models of autism spectrum disorders. Neuropharmacology.

[32] Wan M., Green J., Elsabbagh M., Johnson M., Charman T., Plummer F. (2012). Parent-infant interaction in infant siblings at risk of autism. Res. Dev. Disabil..

[33] Wan M., Green J., Scott J. (2019). A systematic review of parent-infant interaction in infants at risk of autism. Autism: Int. J. Res. Pract..

[34] Courage M., Murphy A., Goulding S., Setliff A. (2010). When the television is on: The impact of infant-directed video on 6- and 18-month-olds’ attention during toy play and on parent-infant interaction. Infant Behav. Dev..

[35] Kirkorian H.L., Pempek T.A., Murphy L.A., Schmidt M.E., Anderson D.R. (2009). The impact of background television on parent-child interaction. Child Dev..

[36] Vandendriessche A., Ghekiere A., Van Cauwenberg J., De Clercq B., Dhondt K., DeSmet A., Tynjala J., Verloigne M., Deforche B. (2019). Does Sleep Mediate the Association between School Pressure, Physical Activity, Screen Time, and Psychological Symptoms in Early Adolescents? A 12-Country Study. Int. J. Environ. Res. Public Health.

[37] Thompson D.A., Christakis D.A. (2005). The association between television viewing and irregular sleep schedules among children less than 3 years of age. Pediatrics.

[38] Chang A.M., Aeschbach D., Duffy J.F., Czeisler C.A. (2015). Evening use of light-emitting eReaders negatively affects sleep, circadian timing, and next-morning alertness. Proc. Natl. Acad. Sci. USA.

[39] Magee C.A., Lee J.K., Vella S.A. (2014). Bidirectional relationships between sleep duration and screen time in early childhood. JAMA Pediatr.

[40] Chindamo S., Buja A., DeBattisti E., Terraneo A., Marini E., Gomez Perez L.J., Marconi L., Baldo V., Chiamenti G., Doria M. (2019). Sleep and new media usage in toddlers. Eur. J. Pediatr..

[41] Paavonen E.J., Raikkonen K., Lahti J., Komsi N., Heinonen K., Pesonen A.K., Jarvenpaa A.L., Strandberg T., Kajantie E., Porkka-Heiskanen T. (2009). Short sleep duration and behavioral symptoms of attention-deficit/hyperactivity disorder in healthy 7- to 8-year-old children. Pediatrics.

[42] Cheng W., Rolls E., Gong W., Du J., Zhang J., Zhang X.Y., Li F., Feng J. (2020). Sleep duration, brain structure, and psychiatric and cognitive problems in children. Mol. Psychiatry.

[43] Vriend J.L., Davidson F.D., Corkum P.V., Rusak B., Chambers C.T., McLaughlin E.N. (2013). Manipulating sleep duration alters emotional functioning and cognitive performance in children. J. Pediatr. Psychol..

[44] Venetsanou F., Kambas A., Gourgoulis V., Yannakoulia M. (2019). Physical activity in pre-school children: Trends over time and associations with body mass index and screen time. Ann. Hum. Biol..

[45] Hinkley T., Brown H., Carson V., Teychenne M. (2018). Cross sectional associations of screen time and outdoor play with social skills in preschool children. PLoS ONE.

[46] Suchert V., Hanewinkel R., Isensee B. (2015). Sedentary behavior and indicators of mental health in school-aged children and adolescents: A systematic review. Prev. Med..

[47] Yang G.Y., Huang L.H., Schmid K.L., Li C.G., Chen J.Y., He G.H., Liu L., Ruan Z.L., Chen W.Q. (2020). Associations Between Screen Exposure in Early Life and Myopia amongst Chinese Preschoolers. Int. J. Environ. Res. Public Health.

[48] He G.H., Strodl E., Liu L., Ruan Z.L., Yin X.N., Wen G.M., Sun D.L., Xian D.X., Jiang H., Jing J. (2019). Teacher’s Type D Personality and Chinese Children’s Hyperactive Behaviors: Moderation Effect of Parental Type D Personality and Mediation Effect of Teacher-Student Relationship. Front. Psychol..

[49] He G.H., Liu L., Strodl E., Ruan Z.L., Jiang H., Jing J., Jin Y., Chen W.Q. (2019). Parental Type D Personality and Children’s Hyperactive Behaviors: The Mediating Role of Parent(-)Child Interactive Activities. Int. J. Environ. Res. Public Health.

[50] Liu L., Fan L., Hou X.Y., Wu C.A., Yin X.N., Wen G.M., Sun D., Xian D.X., Jiang H., Jing J. (2018). Family Childcare Types and Conduct Problem Behaviors in Young Children: The Mediation Role of Caregiver-Child Interaction. Front Pediatr.

[51] Krug D.A., Arick J., Almond P. (1980). Behavior checklist for identifying severely handicapped individuals with high levels of autistic behavior. J. Child Psychol. Psychiatry.

[52] Bravo Oro A., Navarro-Calvillo M.E., Esmer C. (2014). Autistic Behavior Checklist (ABC) and Its Applications. Comprehensive Guide to Autism.

[53] Yang X., Huang Y., Jia M. (1993). Test report of autism behavior checklist in China. Chin. Ment. Health J..

[54] Huang L., Yang G.Y., Schmid K.L., Chen J.Y., Li C.G., He G.H., Ruan Z.L., Chen W.Q. (2020). Screen Exposure during Early Life and the Increased Risk of Astigmatism among Preschool Children: Findings from Longhua Child Cohort Study. Int. J. Environ. Res. Public Health.

[55] Hayes A.F., Aut V.X.X. (2018). Introduction to Mediation, Moderation, and Conditional Process Analysis a Regression-Based Approach Andrew f. Hayes.

[56] Preacher K.J., Hayes A.F. (2008). Asymptotic and resampling strategies for assessing and comparing indirect effects in multiple mediator models. Behav. Res. Methods.

[57] Council on Communications and Media (2016). Media and Young Minds. Pediatrics.

[58] (2019). Guidelines on Physical Activity, Sedentary Behaviour and Sleep for Children under 5 Years of Age.

[59] Canadian Paediatric Society, Digital Health Task Force, Ottawa, Ontario (2017). Screen time and young children: Promoting health and development in a digital world. Paediatr. Child Health.

[60] LeBourgeois M.K., Hale L., Chang A.M., Akacem L.D., Montgomery-Downs H.E., Buxton O.M. (2017). Digital Media and Sleep in Childhood and Adolescence. Pediatrics.

[61] Guerrero M.D., Barnes J.D., Chaput J.P., Tremblay M.S. (2019). Screen time and problem behaviors in children: Exploring the mediating role of sleep duration. Int. J. Behav. Nutr. Phys. Act..

[62] Krupa M., Boominathan P., Ramanan P.V., Sebastian S. (2019). Relationship Between Screen Time and Mother-Child Reciprocal Interaction in Typically Developing Children and Children with Autism Spectrum Disorders. Indian J. Pediatr..

[63] Mendelsohn A.L., Berkule S.B., Tomopoulos S., Tamis-LeMonda C.S., Huberman H.S., Alvir J., Dreyer B.P. (2008). Infant television and video exposure associated with limited parent-child verbal interactions in low socioeconomic status households. Arch. Pediatr. Adolesc. Med..

[64] Detnakarintra K., Trairatvorakul P., Pruksananonda C., Chonchaiya W. (2020). Positive mother-child interactions and parenting styles were associated with lower screen time in early childhood. Acta Paediatr..

[65] Twenge J.M., Hisler G.C., Krizan Z. (2019). Associations between screen time and sleep duration are primarily driven by portable electronic devices: Evidence from a population-based study of U.S. children ages 0-17. Sleep Med..

[66] Xu H., Wen L.M., Hardy L.L., Rissel C. (2016). Associations of outdoor play and screen time with nocturnal sleep duration and pattern among young children. Acta Paediatr..

[67] Healy S., Garcia J., Haegele J. (2020). Environmental Factors Associated with Physical Activity and Screen Time Among Children with and Without Autism Spectrum Disorder. J. Autism Dev. Disord..

[68] Garcia J., Healy S., Rice D. (2016). The Individual, Social, and Environmental Correlates of Physical Activity and Screen Time in Irish Children: Growing Up in Ireland Study. J. Phys. Act. Health.

[69] Xu H., Wen L.M., Hardy L.L., Rissel C. (2016). A 5-year longitudinal analysis of modifiable predictors for outdoor play and screen-time of 2- to 5-year-olds. Int. J. Behav. Nutr. Phys. Act..

[70] Anderson D.R., Subrahmanyam K., Cognitive Impacts of Digital Media Workgroup (2017). Digital Screen Media and Cognitive Development. Pediatrics.

[71] Brown A., Council on Communications and Media (2011). Media use by children younger than 2 years. Pediatrics.

[72] Kuhl P.K. (2007). Is speech learning ‘gated’ by the social brain?. Dev. Sci.

[73] Kuhl P.K., Williams K.A., Meltzoff A.N. (1991). Cross-modal speech perception in adults and infants using nonspeech auditory stimuli. J. Exp. Psychol. Hum. Percept. Perform..

[74] Zlomke K.R., Jeter K. (2020). Comparative Effectiveness of Parent-Child Interaction Therapy for Children with and Without Autism Spectrum Disorder. J. Autism Dev. Disord..

[75] Parlade M.V., Weinstein A., Garcia D., Rowley A.M., Ginn N.C., Jent J.F. (2020). Parent-Child Interaction Therapy for children with autism spectrum disorder and a matched case-control sample. Autism.

[76] Sivertsen B., Harvey A.G., Reichborn-Kjennerud T., Torgersen L., Ystrom E., Hysing M. (2015). Later emotional and behavioral problems associated with sleep problems in toddlers: A longitudinal study. JAMA Pediatr.

[77] Zachor D.A., Vardi S., Baron-Eitan S., Brodai-Meir I., Ginossar N., Ben-Itzchak E. (2017). The effectiveness of an outdoor adventure programme for young children with autism spectrum disorder: A controlled study. Dev. Med. Child Neurol..

[78] Li X., Buxton O.M., Lee S., Chang A.M., Berger L.M., Hale L. (2019). Sleep mediates the association between adolescent screen time and depressive symptoms. Sleep Med..

[79] Exelmans L., Van den Bulck J. (2016). Bedtime mobile phone use and sleep in adults. Soc. Sci. Med..

[80] Luo D., Zhao X., Qu S., Zhang T., Guan H., Guo J. Development of exercise guidelines for preschool children in China. Proceedings of the Abstracts of the 11th National Sports Science Conference.

[81] Li S., Jin X., Wu S., Jiang F., Yan C., Shen X. (2007). The impact of media use on sleep patterns and sleep disorders among school-aged children in China. Sleep.

[82] Liu J., Ge Y. (2015). Psychometric Analysis on Neurotransmitter Deficiency of Internet Addicted Urban Left-behind Children. J. Alcohol. Drug Depend..

[83] Hutton J.S., Dudley J., Horowitz-Kraus T., DeWitt T., Holland S.K. (2019). Associations Between Screen-Based Media Use and Brain White Matter Integrity in Preschool-Aged Children. JAMA Pediatr..

[84] Heffler K.F., Oestreicher L.M. (2016). Causation model of autism: Audiovisual brain specialization in infancy competes with social brain networks. Med. Hypotheses.

[85] Yang W., Xia H., Wen G., Liu L., Fu X., Lu J., Li H. (2015). Epidemiological investigation of suspected autism in children and implications for healthcare system: A mainstream kindergarten-based population study in Longhua District, Shenzhen. BMC Pediatr..

[86] Wang F., Lu L., Wang S.B., Zhang L., Ng C.H., Ungvari G.S., Cao X.L., Lu J.P., Hou C.L., Jia F.J. (2018). The prevalence of autism spectrum disorders in China: A comprehensive meta-analysis. Int. J. Biol. Sci..

[87] Christakis D.A. (2009). The effects of infant media usage: What do we know and what should we learn?. Acta Paediatr..

