# Supplementary material for: Early Electronic Screen Exposure and Autistic-Like Behaviors among Preschoolers: The Mediating Role of Caregiver-Child Interaction, Sleep Duration and Outdoor Activities
Source: Children (Basel). 2020 Oct 28;7(11):200. doi: 10.3390/children7110200 (PMC7692375; doi:10.3390/children7110200)
Supplement: Supplementary file 1 [file children-07-00200-s001.pdf]

children

Supplementary Material:

Table S1. Social-demographic characteristics of the participants by status of Autistic-like behaviors (62 as the cut-off).

Characteristics	Total (*N* = 29461)	Autistic-Like Behaviors No (*N*=29299)	Autistic-Like Behaviors Yes (*N* = 162)	χ^2 / t	*p*-Value
Child's age [mean ± SD (years)]	29461	4.60±0.01	4.44±0.07	2.28	0.023
Maternal age at child's birth [mean ± SD (years)]	29461	27.14±0.02	25.90±0.37	3.31	0.001
Paternal age at child's birth [mean ± SD (years)]	29461	29.71±0.03	28.02±0.39	4.44	<0.001
Child's gender [n (%)]				0.228	0.633
Male	16000	15909 (54.3)	91 (56.2)		
Female	13461	13390 (45.7)	71 (43.8)		
Maternal education level [n (%)]				30.94	<0.001
Junior high school or lower	7367	7298 (24.9)	69 (42.6)		
High school	8604	8557 (29.2)	47 (29.0)		
College	7236	7212 (24.6)	24 (14.8)		
Undergraduate or above	6254	6232 (21.3)	22 (13.6)		
Paternal education level [n (%)]				41.47	<0.001
Junior high school or lower	6076	6011 (20.5)	65 (40.1)		
High school	7954	7911 (27.0)	43 (26.5)		
College	6777	6755 (23.1)	22 (13.6)		
Undergraduate or above	8654	8622 (29.4)	32 (19.8)		
Monthly household income [n (%)]				41.00	<0.001
≤¥5,000 Yuan	4341	4291 (14.6)	50 (30.9)		
¥5,000-10,000 Yuan	7779	7731 (26.4)	48 (29.6)		
¥10,001-15,000 Yuan	5645	5620 (19.2)	25 (15.4)		
¥15,001-20,000 Yuan	4083	4067 (13.9)	16 (9.9)		
＞ ¥ 20,000 RMB	7613	7590 (25.9)	23 (14.2)		
Parental marital status [n (%)]				1.93	0.165
Married	28417	28264 (96.5)	153 (94.4)		
Unmarried/Divorced/ Widowed/Remarried	1044	1035 (3.5)	9 (5.6)		

χ^2: Value for Chi-square test. *t*: Value for Student's *t*-test. SD: Standard Deviation. n (%): Number (proportion).

Table S2. Associations between electronic screen exposure at age 0 to 3 years and autistic-like behaviors in preschoolers (62 as the cut-off).

Screen Exposure at Age 0 to 3 Years	Autistic-Like Behaviors（*N* = 29461）		
	Number of Children	Cases（N%）	AOR（95% CI）
Exposure to electronic screens			
No	7097	27（0.4）	Ref
Yes	22364	135（0.6）	1.441（0.947, 2.193）
Average daily screen time（minutes）			
Never	7097	27（0.4）	Ref
＜30	8271	25（0.3）	0.767（0.444, 1.325）
30-60	6857	35（0.5）	1.224（0.736, 2.034）
60-90	3723	26（0.7）	1.646（0.952, 2.847）
90-120	2303	29（1.3）	2.968（1.739, 5.067）***
＞120	1210	20（1.7）	3.929（2.177, 7.092）***

Adjusted for child's age, child's gender, maternal and paternal age at child's birth, maternal and paternal education level, monthly household income, parental marital status. AOR: Adjusted odds ratio. CI: Confidence intervals. Ref: Reference. *** p<0.001.

Table S3. Associations among average daily screen time, caregiver-child interaction, sleep duration, outdoor activities and autistic-like behaviors (62 as the cut-off).

	Average Daily Screen Time at Age 0 to 3 Years（minutes）	Autistic-Like Behaviors
	β, 95%CI	AOR, 95% CI
Caregiver-child interaction	−0.031（−0.037, −0.026）***	0.461（0.380, 0.558）***
Sleep duration	−0.058（−0.075, −0.040）***	0.871（0.810, 0.937）***
Outdoor activities	−0.253（−0.469, −0.037）*	0.987（0.979, 0.995）**

Adjusted for child's age, child's gender, maternal and paternal age at child's birth, maternal and paternal education level, monthly household income, parental marital status. β: The coefficient of linear regression models. CI: Confidence intervals. AOR: Adjusted odds ratio. * p<0.05; ** p<0.01; *** p<0.001.

Table S4. Mediation effect of average daily screen time on autistic-like behaviors through caregiver-child interaction, sleep duration and outdoor activities (62 as the cut-off).

	**Effect Size (SE)**	**Bootstrapping BC 95% CI**		**Proportion of Indirect Effect**
		**Lower**	**Upper**	
		Direct effects		
	0.300（0.052）	0.198	0.402	
		Indirect effects		
Caregiver-child interaction	0.022（0.004）	0.014	0.032	6.83%
Sleep duration	0.006（0.002）	0.002	0.011	1.96%
Outdoor activities	0.001（0.002）	−0.001	0.006	NS
Total	0.029（0.005）	0.021	0.039	8.81%
		Contrasts		
Caregiver-child interaction vs sleep duration	0.016（0.005）	0.007	0.026	
Caregiver-child interaction vs outdoor activities	0.021（0.005）	0.011	0.032	
Sleep duration vs outdoor activities	0.004（0.003）	−0.001	0.010	

A bootstrapping procedure with 5000 resamples was implemented by the PROCESS macro for SPSS. Indirect effects were estimated with bias corrected confidence intervals (BC 95% CI), which were considered significant if the upper and lower bound of the 95% CI did not straddle zero. NS: Not significant. Adjusted for child's age, child's gender, maternal and paternal age at child's birth, maternal and paternal education level, monthly household income, parental marital status.

Table S5. Comparison of social-demographic characteristics and study variables among population enrolled and excluded.

**Characteristics**	**Total (*N* = 134)**	**Population**		**χ^2 / t**	***p*-Value**
		**Enrolled (*N* = 29461)**	**Excluded (*N* = 134)**		
Child's age [mean ± SD (years)]	29595	4.60±0.88	5.07±0.62	6.27	<0.001
Maternal age at child's birth [mean ± SD (years)]	29591	27.13±4.23	27.51±4.96	1.00	0.317
missing	4		4		
Paternal age at child's birth [mean ± SD (years)]	29523	29.73±4.81	31.44±5.93	2.83	0.005
missing	72		72		
Child's gender [n (%)]				0.04	0.832
Male	16074	16000 (54.3)	74 (55.2)		
Female	13521	13461 (45.7)	60 (44.8)		
Maternal education level [n (%)]				4.29	0.231
Junior high school or lower	7383	7367 (25.0)	16 (36.4)		
High school	8618	8604 (29.2)	14 (31.8)		
College	7243	7236 (24.6)	7 (15.9)		
Undergraduate or above	6261	6254 (21.2)	7 (15.9)		
missing	90		90		
Paternal education level [n (%)]				5.652	0.130
Junior high school or lower	6085	6076 (20.6)	9 (27.3)		
High school	7967	7954 (27.0)	13 (39.4)		
College	6790	6777 (23.0)	13 (9.1)		
Undergraduate or above	8662	8654 (29.4)	8 (24.2)		
missing	101		101		
Monthly household income [n (%)]				14.618	0.006
≤ ¥ 5,000 Yuan	4362	4341 (14.7)	21 (30.0)		
¥ 5,000-10,000 Yuan	7796	7779 (26.4)	17 (24.3)		
¥ 10,001-15,000 Yuan	5659	5645 (19.2)	14 (20.0)		
¥ 15,001-20,000 Yuan	4089	4083 (13.9)	6 (8.6)		
＞ ¥ 20,000 Yuan	7625	7613 (25.8)	12 (17.1)		
missing	64		64		
Parental marital status [n (%)]				390.68	<0.001
Married	28452	28417 (96.5)	35 (51.5)		
Unmarried/Divorced/ Widowed/Remarried	1077	1044 (3.5)	33 (48.5)		
missing	66		66		
Total score of ABC [mean ± SD]	29595	4.90 (10.89)	3.90 (9.26)	−1.064	0.287
Average daily screen time (minutes)				6.627	0.250
Never	7101	7097 (24.1)	4 (3.0)		
＜30	8282	8271 (28.1)	11 (8.2)		
30-60	6884	6857 (23.3)	27 (20.1)		
60-90	3761	3723 (12.6)	38 (28.4)		
90-120	2334	2303 (7.8)	31 (23.1)		
＞120	1233	1210 (4.1)	23 (17.2)		

Continued Table S5. Comparison of social-demographic characteristics and study variables among population enrolled and excluded.

Characteristics	Total (*N* = 134)	Population		χ^2 / t	*p*-Value
		Enrolled (*N* = 29461)	Excluded (*N* = 134)		
Caregiver-child interaction at aged 0–3 years (Index) [mean ± SD]	29595	2.77 ± 0.72	2.67±0.76	−1.178	0.239
Sleep duration at aged 0-3 years (Index) [mean ± SD]	29595	13.20 ± 2.14	13.50±2.45	−1.403	0.161
Outdoor activities at aged 0-3 years (Index) [mean ± SD]	29595	29.85 ± 26.17	27.96±22.50	−0.832	0.405

χ^2: Value for Chi-square test. *t*: Value for Student's *t*-test. SD: Standard Deviation. n (%): Number (proportion).
